# Graph-based representation for identifying individual travel activities with spatiotemporal trajectories and POI data

**DOI:** 10.1038/s41598-022-19441-9

**Published:** 2022-09-21

**Authors:** Xinyi Liu, Meiliu Wu, Bo Peng, Qunying Huang

**Affiliations:** 1 Spatial Computing and Data Mining Lab, Department of Geography, University of Wisconsin-Madison, Madison, 53706 USA; 2 PAII Inc., Palo Alto, 94306 USA

**Keywords:** Psychology and behaviour, Socioeconomic scenarios, Sustainability

## Abstract

Individual daily travel activities (e.g., work, eating) are identified with various machine learning models (e.g., Bayesian Network, Random Forest) for understanding people’s frequent travel purposes. However, labor-intensive engineering work is often required to extract effective features. Additionally, features and models are mostly calibrated for individual trajectories with regular daily travel routines and patterns, and therefore suffer from poor generalizability when applied to new trajectories with more irregular patterns. Meanwhile, most existing models cannot extract features to explicitly represent regular travel activity sequences. Therefore, this paper proposes a graph-based representation of spatiotemporal trajectories and point-of-interest (POI) data for travel activity type identification, defined as Gstp2Vec. Specifically, a weighted directed graph is constructed by connecting regular activity areas (i.e., zones) detected via clustering individual daily travel trajectories as graph nodes, with edges denoting trips between pairs of zones. Statistics of trajectories (e.g., visit frequency, activity duration) and POI distributions (e.g., percentage of restaurants) at each activity zone are encoded as node features. Next, trip frequency, average trip duration, and average trip distance are encoded as edge weights. Then a series of feedforward neural networks are trained to generate low-dimensional embeddings for activity nodes through sampling and aggregating spatiotemporal and POI features from their multihop neighborhoods. Activity type labels collected via travel surveys are used as ground truth for backpropagation. The experiment results with real-world GPS trajectories show that Gstp2Vec significantly reduces feature engineering efforts by automatically learning feature embeddings from raw trajectories with minimal prepossessing efforts. It not only enhances model generalizability to receive higher identification accuracy on test individual trajectories with diverse travel patterns, but also obtains better efficiency and robustness. In particular, our identification of the most common daily travel activities (e.g., *Dwelling* and *Work*) for people with diverse travel patterns outperforms state-of-the-art classification models.

## Introduction

Individual daily travel activities (e.g., work, eating) can be identified as semantics of peoples’ movement trajectories using their GPS records^1,2^ and surrounding geographic context^3^, which is paramount for analyzing and understanding human mobility and urban dynamics, thus benefits smart city development and sustainable urban planning^4–6^. Furthermore, monitoring changes of individual travel patterns can help predict occurrences of specific behaviors, such as alcohol usage relapse, and thus benefit public health studies^7^. Recently, travel activity identification with machine learning models, especially Bayesian Network and Random Forest, receives relatively high evaluation scores^8,9^.

However, existing activity classification schemes mostly rely on hand-crafted features. These features are often complicated and need to be carefully designed and selected for identifying different activity types^10^, which consumes tremendous labors. Moreover, such calibrated models show poor generalizability and receive low inference accuracy when applied to new trajectories. For example, existing models usually fail to detect *Work* activities that can be performed at multiple different locations^11^. The reason is that these models are calibrated with datasets which mainly contain single-location *Work* activities under travel scenarios of certain groups of individuals (e.g., government officials, full-time students). Inherently, one major weakness of previous models is that they lack the capability to explicitly encode regular travel activity sequences (e.g., *Dwelling* $\rightarrow$ *Work* $\rightarrow$ *Dwelling*), which is crucial for differentiating activity types of people with diverse travel patterns under different travel scenarios^12^.

Meanwhile, graph theories, especially with recent advancements of graph neural networks (GNN), are widely used to explore complex relations and interactions within a network^13^. GNN models have been developed in previous studies (e.g., GCN)^14^, where basic undirected graphs are constructed and activity identification is abstracted as node classification. Preliminary experiment results have demonstrated the effectiveness of GNN based models^14^. However, these results are not well evaluated with valid ground truth data. Additionally, the predictive power of graph-based models are not fully exploited by examining more complex graph structures and by integrating geographic context data^14^. Therefore, this study proposes to leverage advanced graph-based models that can embed activity sequence patterns and geographic context, along with traditional spatiotemporal information, in order to identify activity types of people with diverse travel patterns.

Specifically, this study proposes a graph-based representation (Gstp2Vec) based on GraphSAGE^15^ to automatically generate more informative features (i.e., node embeddings) for activity type identification using individual spatiotemporal travel trajectories and their surrounding points-of-interest (POIs). First, activity zones as clusters of individual travel stay points are conceptualized as a graph, with graph nodes denoting activity occurrences, and edges denoting direct trips between activity locations. Then statistics representing spatiotemporal properties of travel footprints (e.g., trip frequencies, average trip duration, distances to the next footprint) and locational POI distributions (e.g., percentage of restaurant POIs) are calculated for each activity node. These statistics are encoded as node features and edge weights, aggregated through neighbor nodes as neighborhood embeddings, and propagated to identify node types via supervised learning. Activity labels are collected from 167 survey participants in early recovery from alcohol use disorders and are grouped into 8 distinct daily travel activity types as the ground truth, including *Dwelling*, *Work*, *Shopping*, *Visiting Others’ Home*, *Public Drink*, *Liquor Store*, *Public Community*, and *Health*. Subsequently, our proposed model is evaluated over the test dataset with precision, recall, and *F*1 score as performance metrics. Furthermore, activity identification results are analyzed by (1) visualizing generated node embeddings, and (2) measuring classification accuracy with varying input statistics (i.e., node features and edge weights) and aggregator architectures (e.g., hidden layer count).

In summary, our contributions are highlighted as follows:

- Weighted directed graphs are first constructed to represent individual travel activity zones as nodes and interconnecting trips as edges.
- Simple statistics of trajectory footprints representing spatiotemporal movement patterns are encoded as node features and edge weights, without designing and transforming complex features. Statistics of surrounding POIs are also leveraged and encoded as node features.
- A novel graph-based representation method is developed to train automatic feature generators (i.e., aggregators) for more effective activity type identification. Our identification of *Dwelling* and *Work* activities for people with diverse travel patterns outperforms previous most popular classification models with better efficiency and robustness. Other activity types (i.e., *Shopping*, *Visiting Others’ Home*, *Public Drink*, *Health*) are also identified with relatively high *F*1 scores.
- Node embeddings are evaluated and visualized to investigate how individual travel activity types are differentiated with our proposed framework. Different input data representing selected node features and edge weights are also evaluated to measure their impact on identifying each distinct activity type. In addition, different aggregator architectures (e.g., neighborhood size) are evaluated to discuss about model performance.

## Related works

Human trajectory analysis has been an important subject in transportation modeling and human mobility studies by helping explain and predict multi-dimensional urban dynamics and guiding urban planning^4,5,14^. Previously, human trajectory analysis mostly relied on data collected from traditional travel surveys^16^, which were tedious and expensive to collect^16^. Nowadays, the development of Information Communication Technology (ICT) enables to leverage extensive data resources (e.g., GPS trajectories, Wi-Fi records, and social media geo-tags), especially in urban settings, which coincides with the requirement of developing smart cities and sustainable urban planning to accommodate the rapid growth of urban size^4,5,16^.

Individual travel activity identification is an important step of human trajectory analysis by adding semantic meanings to personal movement trajectories. Previously, spatial or temporal features of movement trajectories (i.e., spatiotemporal movement patterns) were extracted to represent characteristics of people’s travel behaviors for activity type identification^17^. Spatial patterns often include visit frequencies of historical locations^1^, radius of gyration^3^, and spatial movement scales^1^. Meanwhile, temporal patterns indicate the duration of travel activities and visit frequencies at specific locations within different periods of time (e.g., daytime, weekdays)^1,18^. For example, Isaacman et al.^17^ defined home and work events based on occurring frequencies of individual footprints within typical time windows to identify *Dwelling* and *Work* activities.

Additionally, spatial datasets were integrated with individual movement trajectories to provide geographic context for travel activity identification^19^. In particular, place categories are usually identified as a proxy of activity identification^9,16^ by referring to the classification and distribution of surrounding geographic objects, such as POIs (e.g., restaurant, bar, shopping mall)^3^. Information of these objects is usually collected from additional GIS layers such as Google Maps^20–22^ and specialized POI data^23^. Recently, more open data sources become available and are leveraged, such as Geonames^24^ and OpenStreetMaps (OSM)^25^.

Moreover, as the key component of activity-based modeling^26^, activity sequence patterns (i.e., regular activity sequences such as *Dwelling* $\rightarrow$ *Work* $\rightarrow$ *Dwelling*) were extracted from individual semantic travel trajectories^27^. Travel activity sequences can be conceptualized as activity networks (i.e., motifs)^12^, which are unraveled with network properties such as node distributions, edge degrees, etc. Cornwell^28^ also acknowledged that travel activity sequences can be explained with classical network concepts (e.g., centralization and homophily). These network properties were analyzed in a heuristic way for activity clustering yet not for daily activity type identification. For example, graphs representing tourism-related travel activities were constructed and graph partitioning was applied to detect tourism communities^29^.

Although spatiotemporal and geographic context features perform well in identifying primary activities including *Dwelling*, *Work*, and *Shopping*, effective features (e.g., daily/weekly/monthly visit frequencies of a specific location^1,18^ and the skewness of frequency distributions^30^) need to be carefully designed during the training process for identifying different activities, which is labor-intensive. Additionally, the training and testing results are unstable, varying significantly with unfavorable hyperparameters and different datasets. Furthermore, features explicitly representing activity sequence patterns are not extracted in most existing models, leading to their suboptimal performance in identifying travel activities at irregular locations with inconstant schedules (e.g., work at multiple locations at different time periods)^11^.

Similar to Bayesian Network, graph based models are developed to capture activity sequence patterns. Especially, spatiotemporal graphs have been widely used in skeleton-based recognition algorithms for identifying micro-level human activities (e.g., sitting, clapping) by classifying the entire graphs^31–33^. However, to the best of our knowledge, only a few graph based deep learning models (e.g., GCN) have been explored for identifying individual travel activities (e.g., *Dwelling*, *Work*, *Public Drink*)^13,14^, which are framed as node classification problems^13,14^. Additionally, the classification performance of GCN models is impacted by suboptimal alignments between subspaces of features, graph structure, and ground truth^34^, where any two subspaces provide inconsistent information^34^. Particularly, a misalignment is usually present in graphs with high heterophily (i.e., connected nodes having different class labels and dissimilar features)^35^.

Meanwhile, some GNN models with attention mechanisms (e.g., GraphSAGE^15^) are designed to separate ego-embedding (i.e., a node’s embedding) from the aggregated embeddings of its neighbor nodes^35^, which outperform GCN models in node classification tasks using graphs with relatively high-level heterophily^35^. Furthermore, these graph based representation learning models aim to efficiently generate low-level embeddings for downstream classification tasks, such as predicting user interest in a social network and labeling functions of proteins based on their interactions^15,36,37^. However, graph based representation learning has not been well investigated for travel activity type identification.

## Methods

### Problem formulation

Travel activity zones aggregated from travel trajectory footprints of an individual (*u*) comprise a graph:

1 $$\begin{aligned} G_{u} = (V_{u},E_{u}), \end{aligned}$$

where $V_{u}$ represents a set of graph nodes:

2 $$\begin{aligned} V_{u}=\{v_{u,i}|i \in [0,n]\}, \end{aligned}$$

and each node ($v_{u,i}$) represents an individual representative travel activity zone (i.e., activity node) resulted from aggregating individual travel footprints. Activity nodes of the same individual are connected via a set of graph edges, denoted as:

3 $$\begin{aligned} E_{u}=\{e_{u,j}|j \in [0,m]\}. \end{aligned}$$

Each edge ($e_{u,j}$) represents a directed trip between two end-on activity nodes. General statistics representing spatiotemporal properties of travel trajectories are thus encoded as node features ($X_{v}$) and edge weights ($Y_{e}$). Distributions of surrounding POIs are also encoded as node features. The encoding mechanism is explained in the next two subsections. Each activity node has two types of neighbors, namely in-neighbors ($N_{in}$) and out-neighbors ($N_{out}$). For activity node $v'$ as a neighbor node of activity node *v*,

4 $$\begin{aligned}&{if (v',v) \in E, v' \in N_{in}}, \end{aligned}$$

5 $$\begin{aligned}&{if (v,v') \in E, v' \in N_{out}}. \end{aligned}$$

**Figure 1:**
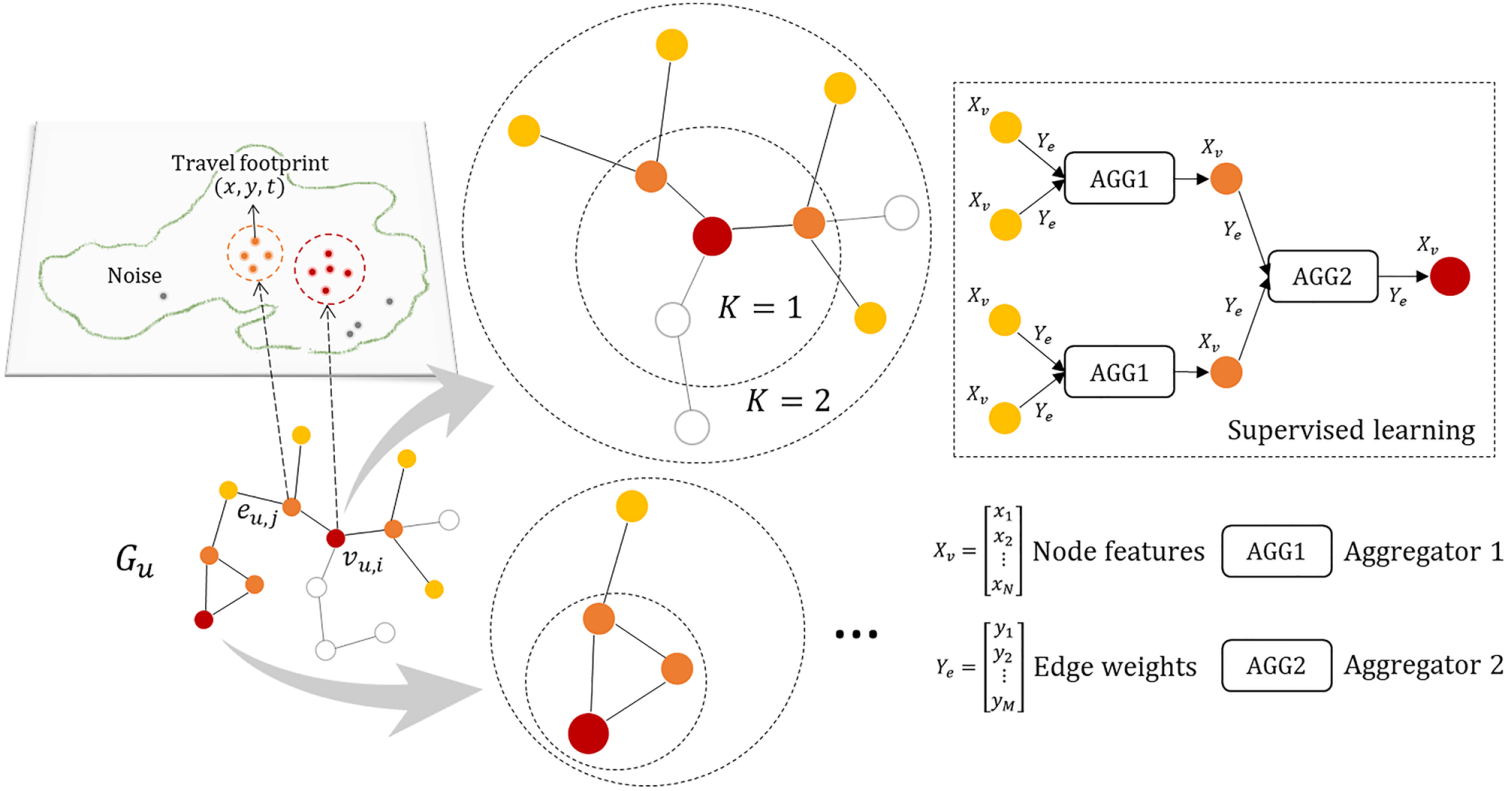
Graph based representation learning for individual travel activity type identification.

Based on the travel activity graph, Gstp2Vec is demonstrated in Fig. 1. Essentially, two sets of fully-connected feedforward neural networks (NN) are created by combining weights with feature embeddings for propagating the information from nodes’ neighbors through the graph structure^15,37^. One set of NNs are wrapped as multihop (i.e., *K*-hop) aggregators for accumulating neighborhood embeddings from sampled neighbor nodes and edges within *K* hops. Specifically, each aggregator function (e.g., *AGG*1, *AGG*2) includes a fully connected NN layer with a nonlinear activation function $\sigma$. Node embeddings (initially node features) and edge weights are concatenated to generate low-dimensional embeddings through the aggregator.

Another set of NNs are built to generate updated node embeddings with the input as node embeddings concatenated with aggregated neighborhood embeddings. Updated node embeddings are treated as predictive representations, which are input into another activation function for inferring travel activity types. This process is called forward propagation^15^, which is iterated over all activity nodes during one epoch for training the model. In this way, information of activity nodes far away from the current one is propagated to it through multihop neighbors and thus contributes to identifying its activity type. Weight matrices and aggregator parameters in the forward propagation are tuned by minimizing graph based cross-entropy loss with an Adam optimizer^38^, thus, making Gstp2Vec a supervised learning model.

### Activity nodes

Individual travel trajectories are represented as sequences of travel footprints, with each footprint representing individual presence at a location and a time point, and denoted as a pair of geographic coordinates with a timestamp. Travel stay points with a speed slower than 1300 m/h^39^ are detected based on spatial adjacency using density-based spatial clustering of applications with noise (DBSCAN)^40^. Activity zones are generated as convex hulls of the detected spatial clusters (i.e., stay regions) so that spatial scopes are specified for the represented travel activities^41^. Then activity zones are used to produce features for activity type identification.

### Node features

The topological relationship between each node and its neighbor nodes, and the distribution of node features on its neighborhood are encoded and propagated to identify the activity type represented by each node. General statistics signifying distribution patterns of footprints on time and space, and distributions of surrounding POIs for each activity zone are calculated and concatenated as node features.

Specifically, the total or average numbers of footprints within each of 24 h on either weekdays or weekends are counted to represent time properties (*t*) of each individual activity zone, where *T* denotes the transpose of a matrix:

6 $$\begin{aligned} t_{weekday}= & {} [n_{1},n_{2},\ldots ,n_{24}]^T, \end{aligned}$$

7 $$\begin{aligned} {\overline{t}}_{weekday}= & {} [{\overline{n}}_{1},{\overline{n}}_{2},\ldots ,{\overline{n}}_{24}]^T, \end{aligned}$$

8 $$\begin{aligned} t_{weekend}= & {} [n_{1}',n_{2}',\ldots ,n_{24}']^T, \end{aligned}$$

9 $$\begin{aligned} {\overline{t}}_{weekend}= & {} [{\overline{n}}_{1}',{\overline{n}}_{2}',\ldots ,{\overline{n}}_{24}']^T, \end{aligned}$$

10 $$\begin{aligned} t= & {} \left[ t_{weekday},{{\overline{t}}_{weekday}}, t_{weekend}, {{\overline{t}}_{weekend}}\right] . \end{aligned}$$

Additionally, average durations spent at an individual activity zone during each date of a week are calculated and concatenated to generate an augmented representation^42^ ($t^{+}$) of temporal patterns:

11 $$\begin{aligned} \overline{\Delta t}_{dow}= & {} \left[\overline{\Delta t}_{1}',\overline{\Delta t}_{2}',\ldots ,\overline{\Delta t}_{7}'\right]^T, \end{aligned}$$

12 $$\begin{aligned} t^{+}= & {} [t,\overline{\Delta t}_{dow}]. \end{aligned}$$

The maximum and average values of elapsed time ($\Delta t_{max}$ and $\overline{\Delta t}$) or distance ($\Delta d_{max}$ and $\overline{\Delta d}$) to the next travel footprint for all footprints within an activity zone are also calculated as spatiotemporal features (*s*):

13 $$\begin{aligned} s=\left[ \Delta t_{max},\overline{\Delta t},\Delta d_{max},\overline{\Delta d}\right] . \end{aligned}$$

To encode POI distribution characteristics, in analogy to natural language processing^43^, each distinct POI feature class (e.g., dormitory, café, bar, hospital, etc.) is considered as a word^44^. All possible POI feature classes are considered as a dictionary, and feature classes of the POIs overlapped with an activity zone are considered as a corpus. A total of 335 distinct POI feature classes (i.e., words) are collected from the OSM dictionary. Then the occurrences of each possible POI feature class are counted for an activity zone to produce a sparse POI feature vector (*p*).

Additionally, 335 POI feature classes are aggregated into 18 distinct place types (e.g., home, eating, education) based on their functionality in urban settings (e.g., café $\rightarrow$ eating)^25^. Then a smaller word dictionary is built to produce a denser vector, which is concatenated with *p* to generate an augmented POI feature vector ($p^{+}$). Next, $p^{+}$ is concatenated with the aforementioned spatiotemporal feature vector to produce a node feature matrix ($X_v$) for each individual activity zone:

14 $$\begin{aligned} {X_v}=\left[ {t^{+}},s,{p^{+}}\right] . \end{aligned}$$

### Edge weights

A trip is defined as the transition from one travel activity (i.e., origin) to another (i.e., destination) for an individual, which usually also indicates the spatial transition of the individual from one location to the other. In our proposed Gstp2Vec framework, trip directions are consistent with edge directions. In addition to trip direction and properties of its end-on activity nodes, trip properties also include statistics measuring individual transitions over space and time, such as travel frequency (*f*), average travel duration (${\overline{t}}$), and average travel distance (${\overline{d}}$), which are encoded as edge weights ($Y_e$).

Specifically, *f* is calculated by counting trip occurrences from every origin activity zone to the corresponding destination zone for each individual by going through all travel footprints within the origin zone. Then ${\overline{t}}$ is measured by averaging the time spent on those trips, and ${\overline{d}}$ is measured by averaging their straight line distances on 2D space. These statistics measuring different aspects of trip properties are concatenated to represent *Y*:

15 $$\begin{aligned} {Y_e}=\left[ f,{\overline{t}},{\overline{d}}\right] . \end{aligned}$$

### Aggregators

**Figure 2:**
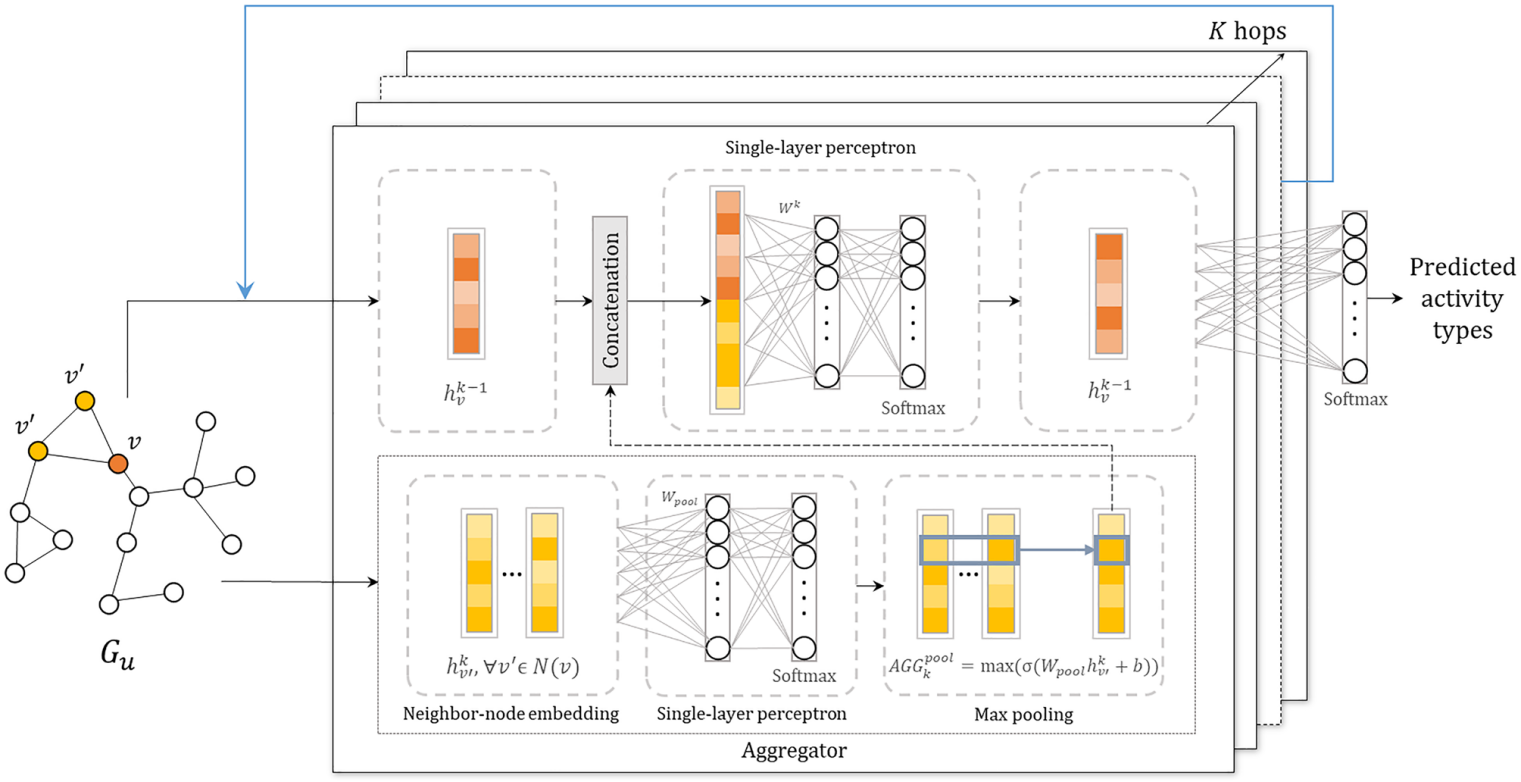
Architecture of aggregators and supervised learning.

As shown in Table 1 and Fig. 2, aggregators (i.e., aggregation functions) in Gstp2Vec accept feature embeddings of sampled neighbor nodes, which are initialized as node features concatenated with their corresponding edge weights. Since neighbor nodes are not ordered by nature in our proposed framework, aggregation functions should be symmetric to be operated on arbitrarily ordered node embeddings. Besides, they need to be simple and trainable^15^. Max pooling aggregator is both symmetric and trainable, and is thus applied in our proposed framework^45^.

**Table 1:** The $k{^{th}} (\forall k \in {1,\ldots ,K})$ aggregator architecture.

Module	Operation	Notation
Input	Node embeddings of sampled neighbor nodes	$h_{{v'}}^{k},\forall {v'} \in N(v),$ $h_{{v'}}^{initial}=\bigg [X_{{v'}},Y_{{e}}\bigg],$ $\forall e \in \bigg \{(v,v'), (v',v)\bigg \}$
Aggregator	Single-layer perceptron with activation function and max-pooling	$AGG_{k}^{pool}=$ $max\bigg (\bigg \{\sigma \bigg (W_{pool}h_{{v'_{i}}}^k+b\bigg ),$ $\forall {v'_{i}}\in N(v)\bigg \}\bigg )$
Output	Neighborhood embedding	$h_{N(v)}^k$

Specifically, a single-layer perceptron is applied as the fully-connected NN inside an aggregator. During every iteration of the forward propagation, a fixed number (e.g., 2) of neighbor nodes are sampled for each activity node. Then the perceptron is applied on the feature embedding matrix of each sampled neighbor node to compute a series of features, and an element-wise maximum value is generated for each computed feature among all sampled neighbor nodes and passed to the current node. In this way, the model effectively captures different aspects of the neighborhood set^15^.

### Supervised learning

**Figure 3:**
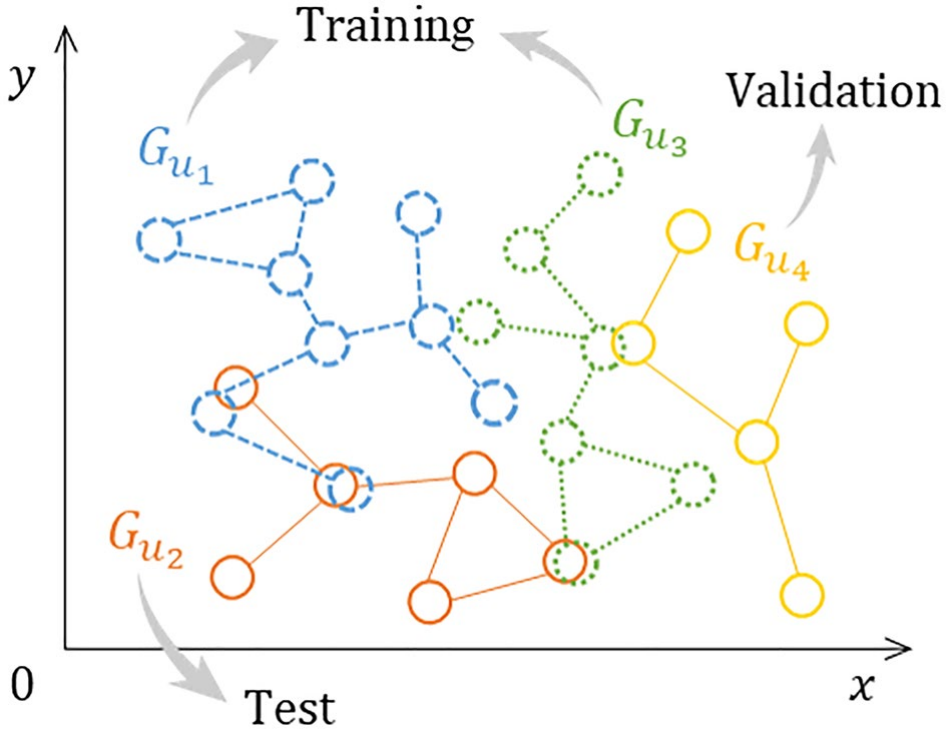
Random split of activity zone graphs into training, validation, and test sets based on the individuals they belong to.

Model weights are tuned iteratively in a manner of end-to-end supervised learning^14,15,31^. First, graphs consisting of activity zones and trips are split into training, validation, and test sets based on individuals (Fig. 3). As such, activity zones of the same individual would not appear in different sets (e.g., both training and test sets). For example, the graph ($G_{u_{2}}$ in Fig. 3) constituted by activity zones of individual $u_{2}$ is divided into the test set, while two other graphs (i.e., $G_{u_{1}}$ and $G_{u_{3}}$) are in the training set and the remaining one (i.e., $G_{u_{4}}$ is in the validation set.

The training process includes two steps, namely forward propagation and parameter learning. Forward propagation (Z in Eq. (16)^46^) first generates node embeddings by concatenating node features with neighborhood embeddings (Fig. 2), which in turn are generated via aggregators as discussed above. Then the concatenated node features are assimilated by a single-layer NN with an activation function, which produces updated node feature embeddings and eventually generates the predictive representations (i.e., $h^{k-1}_v$) (Table 2). Next, another softmax function is applied on the output representations to predict travel activity types via multicategory classification (argmax in Eq. (16)). For parameter learning, graph based cross-entropy loss is applied on the predicted results to tune previous weight matrices.

16 $$\begin{aligned} {Z=f\bigg (h_{v}^{k-1},h_{v'}^{k}\bigg )=argmax\bigg (softmax\bigg (\sigma \bigg (W^k\cdot CONCAT\bigg (h_{v}^{k-1},h_{N(v)}^{k}\bigg )\bigg )\bigg )\bigg )}. \end{aligned}$$

**Table 2:** The architecture of updating node embeddings for nodes in the ${(k-1)}{\text {th}} (\forall k-1 \in {1,\ldots ,K})$ hop.

Module	Operation	Notation
Input	Node embeddings	$h_{v}^{k-1},$ $h_{v}^{initial}=X_{v}$
Aggregator	Aggregation of neighbor nodes	${h_{N(v)}^k=}AGG{_{k}^{pool}}{\bigg (h_{v'}^{k})}$
Fully-connected NN	(1) Concatenation; (2) Single-layer perceptron with softmax activation	$\sigma \bigg (W^k \cdot$ $CONCAT\bigg (h_{v}^{k-1},h_{N(v)}^k\bigg )\bigg )$
Output	Updated node embeddings	$h_v^{{k-1}}$

## Results

### Data

A total of 924,195 travel footprints are collected for the 167 individuals in Madison, WI, who are in early recovery from alcohol use disorders. Each footprint includes a pair of GPS coordinates (i.e., latitude and longitude) and a timestamp. The time span of footprints left by each individual ranges from 3 days to 3 months. Next, 427,891 stay points are detected from these footprints. Then DBSCAN is applied on the stay points, with 50 m as *eps* (i.e., the maximum distance between two points within the same cluster) and 4 as *minPts* (i.e., the minimal points to form a cluster), to generate 10–30 activity zones for each individual.

Next, 2401 travel activity zones with valid ground truth labels (i.e., *non-Others*) are selected. Accordingly, 5 individuals are entirely removed because their activity zones are all labeled as *Others*. Activity zone graphs of the remaining 162 individuals are randomly split into three sets based on individuals (Fig. 3), namely training, validation, and test sets^46^, with a ratio of 8:1:1. As a result, the training, validation, and test sets contain 130, 16, and 16 individuals, respectively. Correspondingly, 130, 16, and 16 graphs are built for each of these three sets. These graphs contain 2401 nodes and 11974 edges in total.

### Model performance

Aggregator functions in the proposed model are fitted on the training set, and evaluated by comparing predicted types of the test set with their actual activity types indicated by place labels as ground truth. The impact caused by different architectures of aggregator functions is discussed in the “Methods” section. Specifically, the training process with random shuffling is conducted on the training set for 10 times^14^ and a confusion matrix is generated each time, of which average percentages of correct predictions are calculated and displayed in Table 3. Additionally, the validation set is tested during the training process for hyperparameter tuning (i.e., hidden layer count, sample size, layer size, dropout rate) and to avoid overfitting^46^.

**Table 3:** Confusion matrix of activity type identification on the test dataset with Gstp2Vec framework.

Real/predicted	*Dwelling*	*Work*	*Shopping*	*V.O.H*	*P.D.*	*P.C.*	*Health*
*Dwelling*	**0.912**	0.009	0	0.05	0.012	0	0.018
*Work*	0.053	**0.632**	0.024	0.116	0.087	0	0.087
*Shopping*	0	0.033	**0.846**	0.051	0.062	0	0.009
*V.O.H.*	0.097	0.063	0.05	**0.713**	0.045	0	0.032
*P.D.*	0	0.041	0.347	0.103	**0.478**	0	0.031
*P.C.*	0	0.054	0.279	0.213	**0.421**	0	0.033
*Health*	0.002	0.13	0.146	0.241	0.061	0	**0.42**

*V.O.H.* Visiting Others’ Home, *P.D.* Public Drink, *P.C.* Public Community.

Maximum ratios of predicted types for each ground truth activity label are in bold.

It can be observed that *Dwelling* activities are the most distinguishable with the proposed Gstp2Vec identification framework. Most *Dwelling* activities are detected with only a few misclassifications, especially as *Visiting Others’ Home*. Additionally, over 80% of *Shopping* activities are successfully identified. The misclassifications of them into other activity types are relatively evenly distributed. Next, around 35% of *Public Drink* activities are misclassified as *Shopping* activities. This is because many *Public Drink* related POIs (e.g., restaurant, café) are located near *Shopping* related POIs (e.g., mall), whereas there are more occurrences of *Shopping* related POIs in our database and we lack supplementary information to precisely differentiate them. *Work* activities are mostly misclassified as *Visiting Others’ Home*, which is likely because either of their identifications relies on both spatiotemporal and POI representations. It is noteworthy that there appear to be no strong properties within the input features for identifying *Public Community* activities, which are defined to occur around various POIs, including park, church, and so on, most (around 80%) of which are misclassified as either *Public Drink* or *Shopping* activities instead. Similarly, there are only a small amount of *Health* related POIs existing in our database, so that many (i.e., 40%) of them are misclassified as either *Shopping* or *Visiting Others’ Home* with more POI instances.

We also compare the proposed Gstp2Vec activity identification framework with traditional heuristic methods and widely used Random Forest (RF) models. Specifically, average values of three evaluation metrics (i.e., precision, recall, and *F*1 score) are calculated for identifying each representative travel activity type with different models (Table 4). The comparison results are analyzed in the discussion section.

**Table 4:** Analysis of the precision (*Pr*), recall (*Re*), and *F*1 score for identifying seven activity types.

Activity type	Classifier	*Pr*	*Re*	*F*1	Avg support
*Dwelling*	Gstp2Vec	**0.773**	0.912	**0.836**	25
	RF	0.762	**0.960**	0.808	
	Heuristic	0.680	0.710	0.690	
*Work*	Gstp2Vec	**0.695**	**0.632**	**0.651**	42
	RF	0.682	0.425	0.522	
	Heuristic	0.220	0.330	0.230	
*Shopping*	Gstp2Vec	0.748	0.846	**0.793**	105
	RF	0.692	**0.908**	0.783	
	Heuristic	**0.910**	0.100	0.180	
*Visiting* *Others’* *Home*	Gstp2Vec	0.524	**0.713**	0.598	42
	RF	**0.545**	0.687	**0.605**	
	Heuristic	0.000	0.000	0.000	
*Public* *Drink*	Gstp2Vec	0.517	0.478	0.489	50
	RF	**0.632**	0.433	**0.512**	
	Heuristic	0.370	**0.820**	0.510	
*Public* *Community*	Gstp2Vec	0.000	0.000	0.000	18
	RF	**0.342**	**0.120**	**0.178**	
	Heuristic	0.004	0.007	0.005	
*Health*	Gstp2Vec	**0.631**	0.420	0.497	36
	RF	0.598	**0.595**	**0.595**	
	Heuristic	0.200	0.140	0.160	

Maximum values of the metric for identifying each activity type are in bold.

### Node embeddings

**Figure 4:**
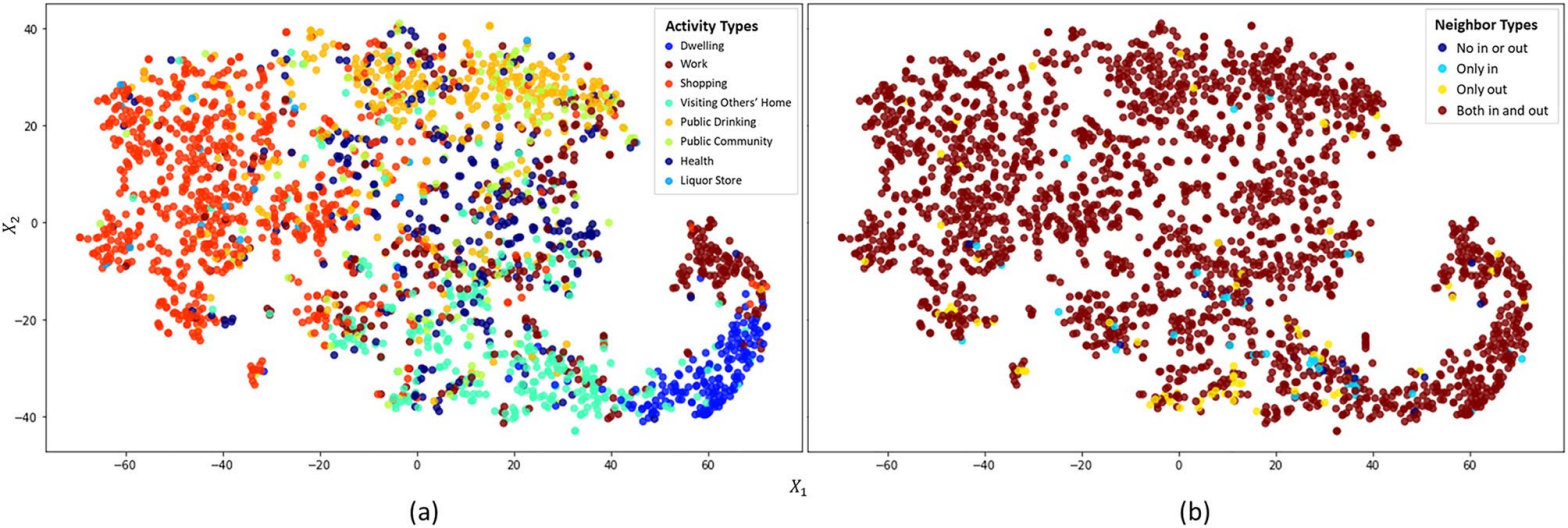
TSNE visualization of node embeddings indicating the distribution of (**a**) activity types, and (**b**) neighbor types for individual travel activity type identification.

This section analyzes travel activity identification results with Gstp2Vec framework through computing and visualizing low dimensional node embeddings. Specifically, node embeddings are generated as activations of the output of forward propagation layer stack. The dimension of node embeddings is thus the same as the size of the last aggregator layer, which is projected as 2D nodes using t-distributed stochastic neighbor embedding (TSNE) for visualization^47^. These nodes are displayed in Fig. 4a with node color indicating identified travel activity types.

From the perspective of node embedding, we also observe that five primary travel activity types are relatively well differentiated, including *Dwelling*, *Work*, *Shopping*, *Visiting Others’ Home*, and *Public Drink*. Meanwhile, *Visiting Others’ Home* tends to be confounded with *Dwelling* activities since they are both located at residential area. When people stay at others’ home, the spatiotemporal properties of their travel footprints could be similar with those at their own home. Additionally, we can see that most activity types are mixed together in the middle of Fig. 4a, especially *Work*, *Health*, *Public Drink*, and *Public Community*, indicating that current input information lacks the capability to differentiate them. Correspondingly, the classifier receives lower evaluation scores in identifying them (Tables 3, 4). Particularly, nodes representing *Health* and *Public Community* activities scatter among the mixture and are hard to identify.

During forward propagation, node features from the previous layer for the node itself, the aggregated in-neighbors, and the aggregated out-neighbors are concatenated in the form of $[X,z_{v,in},z_{v,out}]$. Noticeably, there are four distinct types of directed neighborhoods (Table 5): 1. Having no in or out neighbors with isolated nodes (No In/Out); 2. Only having in-neighbors (Only In); 3. Only having out-neighbors (Only out); 4. Having both in and out neighbors (Both In & Out).

**Table 5:** Node counts based on their neighbor types.

Neighbor type	No In/Out	Only In	Only Out	Both In & Out	Sum
Node count	16	35	75	2275	2401

We also color the nodes based on their neighbor types indicating whether there is in or out neighbors for each node to be classified in the directed graph (Fig. 4b). It shows that most activity nodes without either in or out neighbors are located with a mixture of different activity types. Ideally, every activity node should have at least one in-neighbor and one out-neighbor as individuals travel through every other node from the *Dwelling* node as their daily origin and destination. The missing of either in or out neighbors indicates omissions of trajectory records, which inevitably prevents accurate identification of their represented travel activity types.

### Impact of node features and edge weights

**Figure 5:**
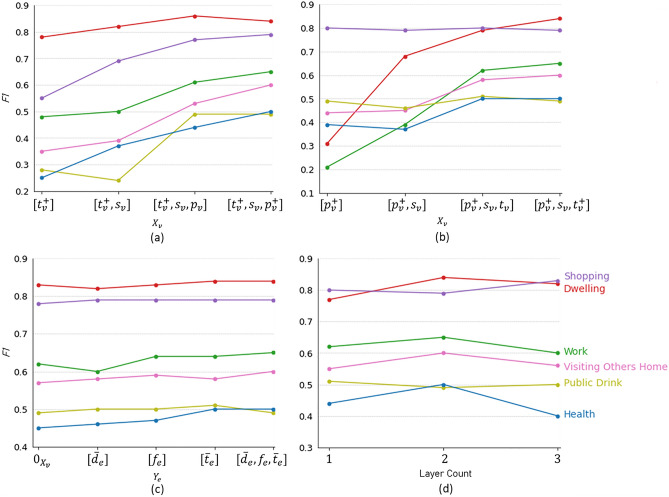
Impact of node features ($X_{v}$): (**a**) POI representations, and (**b**) temporal representations; (**c**) edge weights ($Y_{e}$); and (**d**) hidden layer counts ($N_{L}$) on *F*1 score for identifying six distinct travel activity types.

In this section, we evaluate how node features and edge weights contribute to identifying different individual travel activity types. Figure 5a–c, respectively, show the boosted *F*1 scores brought by POI representations, temporal representations, and edge weights for identifying six distinct travel activity types (i.e., *Dwelling*, *Work*, *Shopping*, *Visiting Others’ Home*, *Public Drink*, and *Health*).

We can see that, even without any POI representations ($p_v$ or $p_v^{+}$) as input node features, the proposed Gstp2Vec framework is able to learn with $t_v^{+}$ and graph structures (e.g., node degrees)^15^ and receives nearly 0.8 as the *F*1 score for identifying *Dwelling* activities. Adding POI representations ($p_{n}$) improves *F*1 scores for identifying all 6 travel activity types, especially those that are highly related to POI types (i.e., *Shopping*, *Public Drink*, and *Health*). Similarly, without input temporal representations ($t_v^{+}$ or $t_v$) as node features, Gstp2Vec receives nearly 0.8 as the *F*1 score for identifying *Shopping* activities. Once $t_v^{+}$ or $t_v$ is added, the *F*1 scores increase for identifying all other activities except for *Shopping*.

Furthermore, introducing edge weights in the model slightly increases *F*1 scores for identifying *Work*, *Visiting Others’ Home*, and *Health* activities, and thus improves the overall accuracy of classifying all 6 activity types. Specifically, Fig. 5c shows that adding a single edge weight (e.g., ${\overline{t}}_e$) can increase the *F*1 score for identifying some activity types (e.g., *Health*) while decrease the *F*1 score for some other types (e.g., *Visiting Others’ Home*). Meanwhile, *F*1 scores for identifying *Dwelling* and *Shopping* activities stay relatively smooth since existing node features consist of less ambiguous information for identifying both of them.

### Comparison of aggregator architecture change

Architectures of aggregator functions are designed to effectively aggregate neighborhood information. The parameter, hidden layer count ($N_{L}$), is defined as the number of hops the aggregator takes along the directed edges to find the neighborhood for each activity node. The model converges with diverse combinations of hidden layer count ($N_{L}$) and hidden feature size ($size_{L}$). Particularly, options of $N_{L}$ can result in different identification accuracy. Other hyperparameters, including hidden feature size, learning rate, batch size, epoch number, and drop-out proportion, are tuned to avoid overfitting.

**Figure 6:**
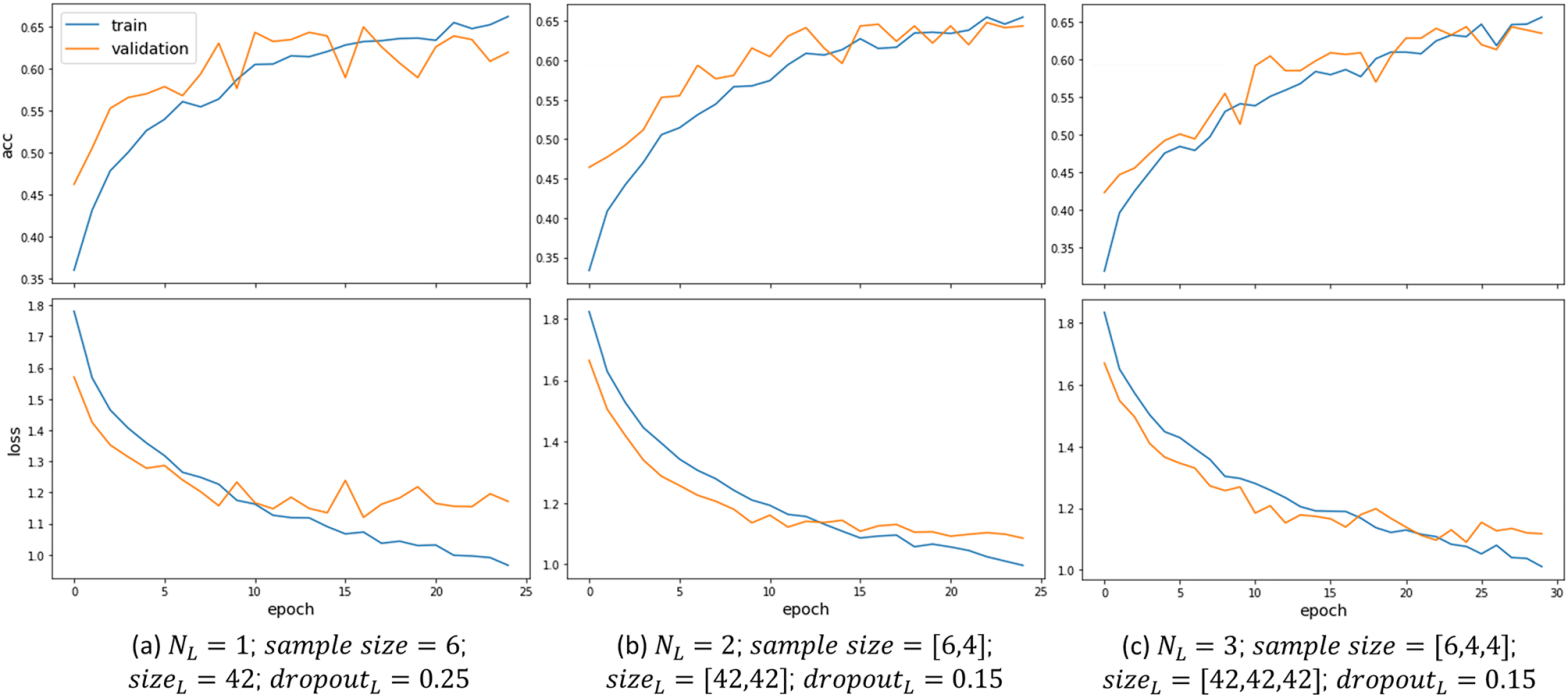
Learning curves of aggregator functions with different hidden layer counts ($N_{L}$) and tuned hyperparameters.

We conduct experiments with $N_{L}$ equal to 1, 2, or 3. It shows that $N_{L}=2$ receives the highest *F*1 score for identifying most activity types, especially *Work*, *Visiting Others’ Home*, and *Health* (Fig. 5d), thus is used in the model for tuning other hyperparameters. Additionally, learning curves of both training and validation datasets are generated with tuned hyperparameters for different options of $N_{L}$ (Fig. 6). It shows that $N_{L}=2$ also obtains the smoothest learning curves. In comparison, $N_{L}=1$ generates the most fluctuant ones.

In terms of other model hyperparameters, our proposed Gstp2Vec learning model reveals less sensibility compared with RF, which is a powerful method for many relevant applications and achieves relatively accurate classifications^48^. The best values of maximum tree number and maximum depth need to be carefully tuned for RF models to make the model converge and avoid overfitting.

## Discussion

Our proposed Gstp2Vec framework successfully extracts effective features automatically for travel activity type identification and achieves the best performance when applied on test trajectories with diverse travel patterns, compared with both heuristic methods and RF models, which were widely applied in previous studies^2,17^. Heuristic methods simply rely on statistical analysis of timestamps attached to footprints within activity zones, where eligible footprints are detected as home or work events and the count or ranking of these events is fitted into a logistic regression model to recognize *Dwelling* and *Work* activities^17^. Furthermore, OSM POIs around activity zones are manually classified into multiple types and the occurrences of each type are counted and compared for identifying the remaining activity types with typical semantic annotation methods^3,20,25^.

Heuristic methods (Heuristic rows in Table 4) receive relatively high evaluation scores (e.g., *F*1 score = 0.690) for identifying *Dwelling* activities and high precision for identifying *Shopping* activities on the same test set as in our proposed model. However, its recall of identifying *Shopping* activities is extremely low as the semantic annotation algorithm prioritizes *Drink* related POIs and activities. Obviously, heuristic methods mostly fail to identify *Work* and *Health* activities as their related POIs are less prominent in the database. Overall, the ambiguity of place categories for revealing travel activity types make them less likely to be correctly identified simply with semantic annotation techniques^49^.

On the other hand, RF models are applied for activity type identification with well-designed hand-crafted features representing spatiotemporal movement patterns and geographic context. The evaluation results (RF rows in Table 4) show that they receive higher scores for identifying all activity types compared with heuristic methods. Especially, RF models reduce classification bias brought by artificial prioritizations of POI types in heuristic methods. For example, the recall of identifying *Shopping* activities is bumped up to around 0.9, whereas its precision keeps around 0.7 to achieve a high *F*1 score as 0.78. Additionally, RF models are able to integrate spatiotemporal movement patterns with geographic context features to successfully identify most of *Visiting Others’ Home* activities. In this way, RF also receives the highest evaluations scores for identifying both *Public Community* and *Health* related activities.

In comparison, our proposed method receives the highest *F*1 scores for identifying primary travel activity types compared with the other two models, including *Dwelling*, *Work*, and *Shopping*. Especially, *F*1 scores for identifying *Dwelling* and *Work* activities are boosted compared with RF models, which means that Gstp2Vec automatically distills additional effective features besides the hand-crafted ones through training its two-hop feature aggregators. However, *Health* activities are identified with relatively low evaluation scores (i.e., $F1 < 0.5$) and no *Public Community* activities are successfully detected (i.e., $F1 = 0$). This is because only limited instances of these activity types exist in our case study dataset, so that their distinguishing features are not evident enough to be captured by the graph learning model, which typically performs well on large datasets.

It is worth noting that *Work* activities are identified with relatively low evaluation scores compared with some previous benchmarking works^2^. However, the high evaluation scores in previous studies is challenged by their sampling bias. In our dataset, people show irregular spatiotemporal travel patterns. For example, many of them commonly conduct non-*Work* activities (e.g., eating, drink) during work hours or work at multiple locations during irregular time slots (e.g., weekend, fragmented time slots on weekdays). Generally, as people’s work schedules become more flexible and diverse^50^, previous best-practice models and data size can hardly generate effective features to capture such irregular movement patterns and to accurately (e.g., recall $\approx$ 0.8^2^) identify *Work* activities.

## Conclusion

With the development of location-based services, a large amount of individual daily travel trajectories can be collected via GPS installed on portable mobile devices, enabling the investigation of semantic patterns of individual daily travel activities. However, previous studies often fail to identify travel activity types for a wide range of population with diverse travel patterns. Additionally, activity type identification models with high evaluation scores mostly rely on labor-intensive engineering work to manually design optimal features for each distinct activity type. Furthermore, instead of conducing rigorous travel surveys to collect ground truth data for training activity type classifiers, previous methods often leverage place types annotated by volunteers on open source platforms. Thus, these methods suffer from the bias of human subjectivity and the ambiguity of place categories.

In response, the Gstp2Vec framework proposed by this study enables automatic generation of informative features via encoding and aggregating general statistics representing spatiotemporal movement patterns and locational POI distributions as node features, along with spatiotemporal patterns as edge weights, in a weighted directed graph. To the best of our knowledge, this work is the first to leverage graph based representation learning models for individual travel activity identification. When applied to identifying travel activities of people with diverse travel activity patterns, our proposed model outperforms previous models by receiving higher precision and recall for identifying primary activity types including *Dwelling* and *Work*, and receiving relatively high *F*1 scores for identifying other POI-related activity types including *Shopping*, *Visiting Others’ Home*, and *Public Drink*.

## Data Availability

The OSM landuse and POI datasets analyzed during the current study can be publicly retrieved from the official OSM website. The preprocessed GPS trajectory data can be made available from the corresponding author on a reasonable request.

## Code availability

Related source codes are published on https://github.com/XinyiHolly/Gstp2Vec-TravelActivityClassification.
